# Case Report: A case of aural myiasis in an elderly bedridden patient with newly diagnosed Parkinson’s syndrome

**DOI:** 10.3389/fsurg.2025.1730434

**Published:** 2026-01-06

**Authors:** Bingyan Hao, Zhen Sun, Caini Peng, Kaijie Zhong, Xianlu Zhuo

**Affiliations:** 1 Department of Otorhinolaryngology, Clinical Medical College of Guizhou Medical University, Guizhou, Guiyang, China; 2 Department of Otorhinolaryngology Head and Neck Surgery, Affiliated Hospital of Guizhou Medical University, Guizhou, Guiyang, China

**Keywords:** aural myiasis, case report, cholesteatoma, myiasis, Parkinson's disease

## Abstract

Myiasis, an infestation of living vertebrates by dipteran larvae, is relatively uncommon in otorhinolaryngology. Chronic otitis media and other ear pathologies are significant predisposing factors. This article presents the case of an 89-year-old bedridden female patient who presented with right ear discharge and visible maggots. Following mastoid exploration and systemic antibiotic therapy, her clinical symptoms improved. This case highlights the increased susceptibility of elderly, bedridden patients to aural myiasis.

## Introduction

Myiasis, derived from the ancient Greek word “myia” (meaning fly), is defined as the infestation of live humans or animals by fly larvae (1). Due to improved living standards, human myiasis has become less prevalent. Within otorhinolaryngology, aural myiasis remains a rare clinical entity (2). Predisposing factors include chronic ear infections, poor personal hygiene, exposure to contaminated water, immunocompromised states, neglected children, advanced age, and intellectual disability (3). Diagnosis is mostly made by history and clinical examination, though further investigations are warranted if middle ear extension is suspected (4). While most cases respond well to topical treatment (5), some may require surgical intervention (6). This article presents a case of aural myiasis in a long-term bedridden elderly patient, emphasizing the critical need for vigilance in vulnerable populations.

## Case report

**Figure 1:**
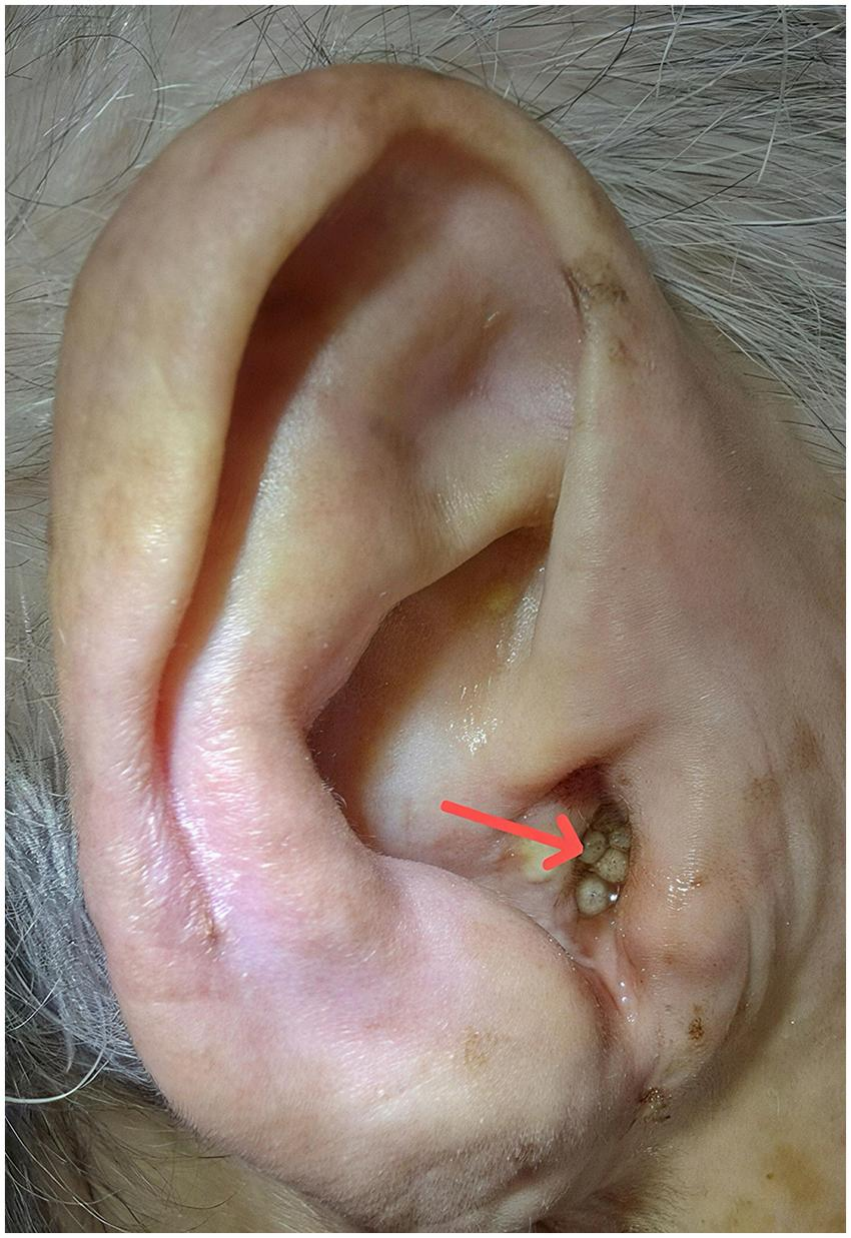
Mobile larvae completely obstructing right external ear canal (arrow).

An 89-year-old female patient was admitted to the hospital on September 22, 2025, following the discovery of maggots in her right external auditory canal seven hours prior. The patient's family members noticed maggots wriggling at the external auditory meatus of the patient's right ear. No definite otalgia or aural pruritus was reported, and the infestation was first observed by family members. The patient had been bedridden at home for the past year. Before becoming bedridden, the patient was largely independent in daily activities. During the bedridden period, the patient's daily care and personal hygiene were primarily provided by family members. The right external auditory canal orifice is occluded by multiple living white maggots (Figure 1), and impacted oily cerumen is present in the left external auditory canal. High-resolution temporal bone computed tomography (CT) demonstrated soft tissue density in the right external auditory canal, bilateral cholesteatoma, and absence of the right ossicles. Hearing tests indicated profound deafness in the right ear. According to the patient's family, there was no known history of chronic otitis media, persistent otorrhea, or previously diagnosed tympanic membrane perforation prior to the current presentation. The patient had no known history of diabetes, vascular disease, malignancy, or chronic immunosuppression. The patient had not been receiving any long-term medications and was not taking antiparkinsonian drugs prior to admission.

**Figure 2:**
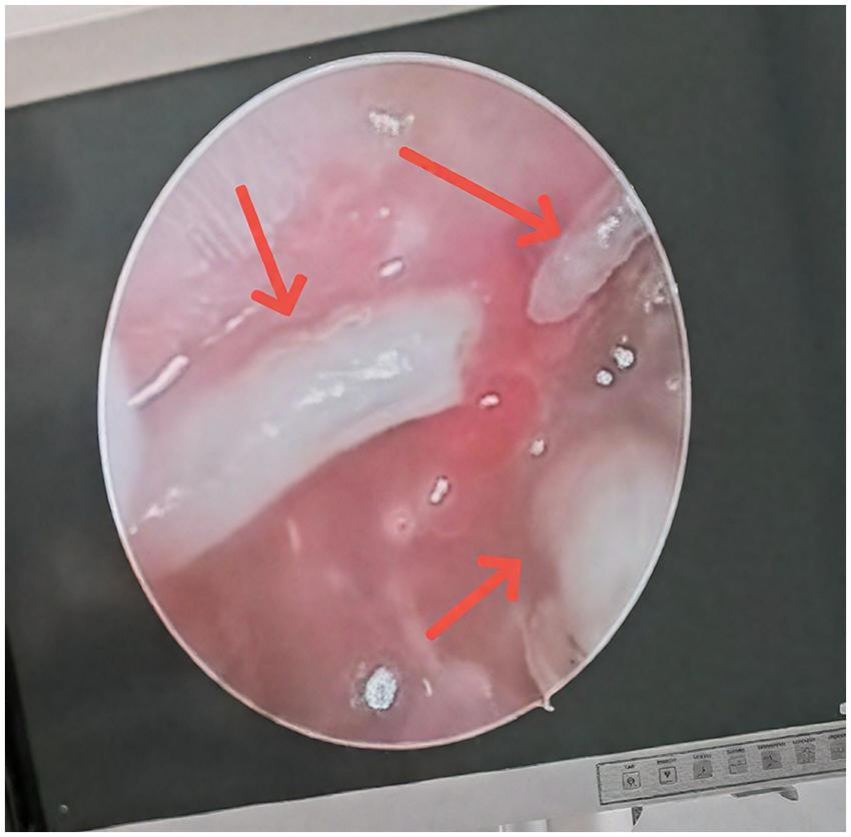
Live maggots in the external ear canal by endoscopic examination (arrow).

During hospitalization, the patient presented with lethargy, unresponsiveness, slowed reactions, and increased muscle tone in all four limbs. A neurological evaluation subsequently established a first-time diagnosis of Parkinson's syndrome. Under otoscopic examination, multiple maggots were observed crawling within the ear canal (Figure 2). After removal of six maggots, swelling of the right external auditory canal and perforation of the right tympanic membrane were identified. The external auditory canal was repeatedly irrigated with iodine brine and normal saline. The patient subsequently received combined topical and systemic antibiotic therapy, including Levofloxacin ear drops and intravenous Cefuroxime (0.75 g every 8 hours for 5 days). Following irrigation and antibiotic therapy, the patient showed partial clinical improvement, with a reduction in visible inflammation and cessation of further maggot emergence. However, swelling of the external auditory canal and signs of tympanic membrane perforation persisted, and imaging continued to show soft-tissue density in the middle ear and mastoid region, raising concern for residual disease. Unfortunately, the patient's family declined morphological identification of the maggots, thus precluding species determination.

The patient presented with a tympanic membrane perforation complicated by middle ear cholesteatoma. Given the suspicion of residual lesions in the middle ear, a right mastoid exploration was performed on September 24, 2025, to prevent potential intracranial and extracranial complications associated with these residual lesions, after carefully ruling out any surgical contraindications. During the operation, a right tympanic membrane perforation was found, and no maggots were detected. The tympanic membrane was lifted, and exploration revealed the absence of the incus and malleus; only the stapes remained in the tympanic cavity, with edema of the middle ear mucosa. Postoperative pathological examination of the specimen revealed inflammatory tissue. The patient was followed for 2 months after surgery. Follow-up otoscopic examinations showed complete epithelialization of the tympanic membrane and a well-healed mastoid cavity. No residual larvae, recurrent otorrhea, or secondary infections were observed during the follow-up period. The patient's hearing in the right ear remained at the preoperative level of profound loss, with no further deterioration. No postoperative complications were recorded.

## Discussion

Flies can deposit eggs and/or live larvae in and around natural body orifices (7). Myiasis is classified based on the affected anatomy, including cutaneous myiasis, myiasis of external orifices (nose, paranasal sinuses, and external ear), or internal organs, and myiasis invading head cavities (8). In mammals, including humans, dipteran larvae can feed on living or necrotic tissues, bodily fluids, or ingested food, potentially causing extensive infestations (9). The extent of infection depends on the fly species, host, the site of infestation, and the host's immune response (2). Infestation of the external and/or middle ear by maggots/larvae is termed aural myiasis (4).

Middle ear cholesteatoma is characterized by a mass of keratinized squamous epithelial cells within the tympanic and mastoid cavities, accompanied by subepithelial connective tissue and accumulated keratin debris, often with an inflammatory response (10). The resulting otitis media can lead to chronic purulent ear discharge, frequently associated with a foul odor. Persistent discharge may also cause local skin necrosis and tympanic membrane perforation. Although no clear otorrhea had been noted before admission, the underlying cholesteatoma likely contributed to retention of secretions and odor formation, which in turn facilitated fly attraction and aural myiasis. A review suggests that the unpleasant odor emanating from the ear canal can attract female flies, prompting them to deposit larvae, thereby leading to aural myiasis. Thus, chronic otitis media and other ear pathologies are significant predisposing factors (11).

Patients mostly present themselves with sensation of foreign substance in the ear, aural itching, pain, bleeding, tinnitus, hearing loss, and vertigo (12). In our case, the patient had otitis media but did not complain of any symptoms, most likely due to cognitive impairment caused by Parkinson's disease, which delayed diagnosis and treatment. Otoscopic examination may reveal maggots, inflammation, and swelling of the ear canal, and sometimes tympanic membrane perforation (13). Documented complications include ossicular destruction leading to hearing loss (14), deafness, and intracranial penetration causing potentially fatal meningitis (15).

Diagnosis of myiasis is typically straightforward, based on clinical findings alone. Currently, there are no established clinical practice guidelines for treating this condition. Directly killing maggots in the ear can make them difficult to locate, and retained dead maggots may provoke a foreign body reaction. Initial treatment primarily involves maggot removal via simple aural suction, supplemented with topical and/or oral antibiotic coverage (8). Various agents, including 70% ethanol, chloroform, normal saline, urea, oil drops, dextrose, creatine, iodine saline, and topical ivermectin, have been used for ear canal irrigation to eliminate residual larvae (16).

For aural myiasis accompanied by otorrhea extending to the middle ear, or in cases of residual disease, extensive surgical debridement, including mastoidectomy followed by reconstruction, is recommended, along with systemic antibiotic therapy.

In this case, the patient presented with concomitant middle ear cholesteatoma and bilateral oily cerumen secretions. The combination of cholesteatoma discharge and oily cerumen created a retention effect, impeding normal drainage. The accumulated secretions emitted odors that attracted flies, leading to larval deposition and subsequent aural myiasis. The patient's delayed symptom reporting was likely due to cognitive impairment associated with Parkinson's syndrome, which postponed diagnosis and treatment. Although the patient was not immunocompromised in a medical sense, advanced age and prolonged bedridden status may have contributed to reduced local defense mechanisms of the external auditory canal.

The presence of tympanic membrane perforation, coupled with middle ear cholesteatoma, necessitated mastoid surgery for thorough debridement and removal of maggots from the sinus opening and mastoid cavity, thereby preventing potential intracranial and extracranial complications from residual lesions.

Compared with previously reported cases of aural myiasis, this case presents several distinctive features. A systematic review and multiple case reports have shown that chronic suppurative otitis media with overt, foul-smelling otorrhea and poor hygiene are among the most frequent predisposing conditions for aural myiasis (11, 17, 18). In particular, many patients described in the literature had long-standing ear discharge and neglected chronic ear disease in association with low socioeconomic status or suboptimal living conditions (17, 18). Furthermore, several authors have emphasized that aural myiasis often occurs in debilitated or cognitively impaired individuals—including patients with Alzheimer's disease or other mental deficits—who depend on caregivers for daily hygiene and medical care (18, 19). In contrast, our patient was an extremely elderly, bedridden individual receiving home-based care by family members rather than institutional care, highlighting that aural myiasis can also arise in the context of home long-term care when regular ear inspection and hygiene are insufficient. Another distinctive aspect of this case is that Parkinson's syndrome with cognitive slowing was newly diagnosed during this admission, and, although the patient was not classically immunocompromised, her neurocognitive impairment likely contributed to delayed symptom recognition and reporting. Finally, while most published cases are managed conservatively, some reports describe middle-ear or mastoid extension requiring surgical intervention (20). In our case, mastoid exploration allowed simultaneous management of underlying cholesteatoma, exclusion of residual disease, and resulted in good anatomic healing without recurrence during follow-up.

Beyond its clinical implications, aural myiasis in a dependent, bedridden elderly patient also raises concerns regarding the possibility of neglect or suboptimal caregiving. Myiasis has been recognized in the forensic and medicolegal literature as a potential indicator of inadequate hygiene, insufficient supervision, or delayed recognition of symptoms in vulnerable individuals who rely on others for daily care. Bugelli et al. emphasized that myiasis may serve as a marker of neglect in living or deceased individuals, particularly when occurring in patients with limited autonomy or cognitive impairment (21). Although our case is purely clinical and not forensic in nature, the occurrence of aural myiasis in this patient highlights the importance of optimizing caregiving practices, maintaining adequate hygiene, and ensuring regular inspection of vulnerable patients to prevent such complications.

Another important limitation of this case is the absence of morphological identification of the larvae. Because the patient's family declined specimen collection, the species of fly responsible for the infestation could not be determined. This lack of entomological characterization restricts the ecological and epidemiological inferences that can be drawn from the case, as different fly species vary in their habitat preferences, life cycles, and medical relevance. Future similar cases would benefit from proper collection, preservation, and morphological—and when feasible, molecular—identification of larvae to enable accurate species determination and enhance understanding of local epidemiological patterns.

Poor hygiene is likely the most significant risk factor for human myiasis (22). Management of myiasis should prioritize prevention through maintaining proper hygiene, improving living conditions, and actively treating external auditory canal or middle ear diseases. For bedridden or cognitively impaired individuals, additional preventive measures are recommended. These include regular inspection and cleaning of the external auditory canal, maintaining scalp and ear hygiene, timely management of cerumen impaction, and ensuring adequate environmental sanitation to minimize fly exposure. Caregivers should be educated to recognize early changes such as new-onset ear discharge, foul odor, or unexplained irritability, particularly in patients with impaired ability to report symptoms. These measures can significantly reduce or eliminate opportunities for flies to enter and lay eggs.

## Data Availability

The original contributions presented in the study are included in the article/Supplementary Material, further inquiries can be directed to the corresponding author.

## References

[1] Mengi E Demirhan E Arslan IB. Aural myiasis: case report. North Clin Istanb. (2014) 1(3):175–7. 10.14744/nci.2014.96967 28058327 PMC5175039

[2] Yuca K Caksen H Sakin YF Yuca SA Kiriş M Yilmaz H Aural myiasis in children and literature review. Tohoku J Exp Med. (2005) 206(2):125–30. 10.1620/tjem.206.125 15888968

[3] Ata N Güzelkara F. Aural myiasis in an infant. J Craniofac Surg. (2017) 28(1):e89–90. 10.1097/SCS.0000000000003272 27922960

[4] Al Jabr I. Aural myiasis, a rare cause of earache. Case Rep Otolaryngol. (2015) 2015:219529. 10.1155/2015/219529 26380140 PMC4561331

[5] Jervis-Bardy J Fitzpatrick N Masood A Crossland G Patel H. Myiasis of the ear: a review with entomological aspects for the otolaryngologist. Ann Otol Rhinol Laryngol. (2015) 124(5):345–50. 10.1177/0003489414557021 25358614

[6] Panu F Cabras G Contini C Onnis D. Human auricolar myiasis caused by wohlfartia magnifica (schiner) (Diptera: sarcophagidae): first case found in sardinia. J Laryngol Otol. (2000) 114(6):450–2. 10.1258/0022215001905814 10962679

[7] Cook DF Voss SC Dadour IR. The laying of live larvae by the blowfly calliphora varifrons (Diptera: calliphoridae). Forensic Sci Int. (2012) 223(1–3):44–6. 10.1016/j.forsciint.2012.07.015 22921421

[8] Wang Y Sun Y Kong W Wang Y. Aural myiasis: a case report and literature review. Ear Nose Throat J. (2022) 101(7):430–2. 10.1177/0145561320966072 33048587

[9] Noutsis C Millikan LE. Myiasis. Dermatol Clin. (1994) 12(4):729–36. 10.1016/S0733-8635(18)30136-0 7805302

[10] Yung M Tono T Olszewska E Yamamoto Y Sudhoff H Sakagami M EAONO/JOS joint consensus statements on the definitions, classification and staging of middle ear cholesteatoma. J Int Adv Otol. (2017) 13(1):1–8. 10.5152/iao.2017.3363 28059056

[11] Rodríguez-Ruiz MT Acosta AM Cifuentes-Cardozo E Chirveches MA Rosselli D. Otomyiasis: systematic review. Int Arch Otorhinolaryngol. (2019) 23(1):104–9. 10.1055/s-0037-1617427 30647793 PMC6331295

[12] Tbini M Jaafoura H Ghabi M Chebil E Bensalah M. Otomyiasis caused by Musca domestica in a child: a case report. Int J Surg Case Rep. (2022) 94:107108. 10.1016/j.ijscr.2022.107108 35468383 PMC9046598

[13] Mallik P Sinha V Jha S Swani J Maniya N Yadav S. A clinico-etiological study of aural myiasis. Indian J Otol. (2019) 25(4):180–3. 10.4103/indianjotol.INDIANJOTOL13117

[14] Rana AK Sharma R Sharma VK Mehrotra A Singh R. Otorhinolaryngological myiasis: the problem and its presentations in the weak and forgotten. Ghana Med J. (2020) 54(3):173–8. 10.4314/gmj.v54i3.8 33883762 PMC8042792

[15] Uzun L Cinar F Beder LB Aslan T Altintas K. Radical mastoidectomy cavity myiasis caused by wohlfahrtia magnifica. J Laryngol Otol. (2004) 118(1):54–6. 10.1258/002221504322731655 14979975

[16] Nayak SP Reddy YL Patil PH Harugop AS Narasimman DB Neema K. Importance of an ent surgeon in maggot removal, improper attempt leads to deadly complications: a case report. Indian J Otolaryngol Head Neck Surg. (2023) 75(2):1204–6. 10.1007/s12070-022-03466-7 37275076 PMC10234996

[17] Övet G Tezer MS Alataş N Kocacan FN. Aural myiasis in a patient with chronic otitis media. Turkish Arc Otolaryngol. (2012) 50:5–7. 10.5152/toa.2012.02

[18] Bayindir T Miman O Miman MC Atambay M Saki CE. Bilateral aural myiasis (wohlfahrtia magnifica): a case with chronic suppurative otitis media. Turkiye Parazitol Derg. (2010) 34(1):65–7. 20340092

[19] Werminghaus P Hoffmann TK Mehlhorn H Bas M. Aural myiasis in a patient with Alzheimer’s disease. Eur Arch Otorhinolaryngol. (2008) 265(7):851–3. 10.1007/s00405-007-0535-2 18030484

[20] Rummens E Van der Mieren G Van Rompaey V Piessens P Somville F. Aural myiasis: a case report on a rare entity. Cureus. (2020) 12(9):e10617. 10.7759/cureus.10617 33123431 PMC7584293

[21] Bugelli V Tarozzi I Galante N Bortolini S Franceschetti L. Review on forensic importance of myiasis: focus on medicolegal issues on post-mortem interval estimation and neglect evaluation. Leg Med (Tokyo). (2023) 63:102263. 10.1016/j.legalmed.2023.102263 37126932

[22] Francesconi F Lupi O. Myiasis. Clin Microbiol Rev. (2012) 25(1):79–105. 10.1128/CMR.00010-11 22232372 PMC3255963

